# Advances in the management of Barrett’s esophagus and early esophageal adenocarcinoma

**DOI:** 10.1093/gastro/gov048

**Published:** 2015-10-19

**Authors:** Ajaypal Singh, Amitabh Chak

**Affiliations:** Division of Gastroenterology and Hepatology, University Hospitals Case Medical Center, Cleveland, OH, USA

**Keywords:** Barrett’s esophagus, early esophageal adenocarcinoma, endoscopy

## Abstract

The incidence of esophageal adenocarcinoma (EAC) has markedly increased in the United States over the last few decades. Barrett’s esophagus (BE) is the most significant known risk factor for this malignancy. Theoretically, screening and treating early BE should help prevent EAC but the exact incidence of BE and its progression to EAC is not entirely known and cost-effectiveness studies for Barrett’s screening are lacking. Over the last few years, there have been major advances in our understanding of the epidemiology, pathogenesis and endoscopic management of BE. These developments focus on early recognition of advanced histology and endoscopic treatment of high-grade dysplasia. Advanced resection techniques now enable us to endoscopically treat early esophageal cancer. In this review, we will discuss these recent advances in diagnosis and treatment of Barrett’s esophagus and early esophageal adenocarcinoma.

## Background

The incidence of esophageal adenocarcinoma (EAC) has increased six- to seven-fold from 1975 to 2006 [1–3]. Barrett’s esophagus (BE) is the most significant known risk factor for esophageal adenocarcinoma. The major challenge in the management of BE is the asymptomatic nature of this condition and the fact that more than half of short-segment BE patients do not have any reflux symptoms. Cancer develops through a sequence of genetic and epigenetic changes that lead to activation of oncogenes and silencing of tumor suppressor genes, with progression from metaplasia through dysplasia to adenocarcinoma. Historically, endoscopic surveillance of BE has been focused on detection of dysplasia and early cancer. Recent advances have now led to an expanding role for endoscopy with the focus on early detection and endoscopic treatment of high-grade dysplasia and early neoplasia. The diagnostic—as well as treatment—options for Barrett’s esophagus and early esophageal cancer have undergone remarkable changes over the last decade, due to better understanding of the disease pathogenesis and advances in endoscopic imaging and therapy. In this review, we will discuss these advances.

Barrett’s esophagus is defined as the presence of metaplastic columnar epithelium in the tubular esophagus above the gastro-esophageal junction (defined by the proximal extent of the gastric folds). This definition involves both endoscopic and histological criteria. Studies have shown that intestinal metaplasia is associated with increased risk of malignancy, but the data for cardia-type metaplasia is not so definitive; hence, the presence of intestinal-type epithelial metaplasia is required for diagnosis in the United States [4].

Even though BE is the most significant known risk factor for esophageal adenocarcinoma, the majority of patients with a new diagnosis of EAC do not have a prior diagnosis of BE. It is unknown whether this is due to large burden of undiagnosed BE in the community or because BE is not the only precursor of EAC. The incidence and prevalence of BE has been an issue for debate, with various studies showing an incidence of 0.5–2% in asymptomatic patients and a slightly higher incidence of 5–10% in patients with symptomatic reflux [5–8].

## Advances in screening for barrett’s esophagus: diagnosing intestinal metaplasia

Screening for BE is a controversial issue, given the fact that the prevalence of BE in the general population is low, the incidence of EAC in patients with BE is 0.1–0.3% per year [9, 10], and cost-effective studies to prove the benefit for screening are not available. With better understanding of the epidemiology of BE and EAC, we can now identify an at-risk population. The American Gastroenterological Association (AGA) recommends that screening be considered in adults above 50 years of age with multiple risk factors while, due to lack of data, the American Society of Gastrointestinal Endoscopy (ASGE) does not recommend routine screening [4, 11]. The ASGE’s position is that Caucasian men above 50 years of age and with risk factors such as smoking or abdominal obesity along with symptoms of chronic reflux or heartburn should be considered for Barrett’s screening; however, studies have failed to show that conventional endoscopy is cost-effective in BE screening, due to the requirement for sedation and longer procedure times. Efforts are being made to minimize these costs and to develop screening techniques that can be used in the clinic setting without the need for sedation.

### Transnasal endoscopy

Transnasal endoscopy (TNE) is performed without sedation, using ultra-thin endoscopes advanced through the nares, with application of topical anesthetic only. It can be performed in the office setting and is a promising tool that avoids the costs associated with diagnostic endoscopy and sedation. In 2002, Saeian *et al*.* showed* for the first time that unsedated TNE was comparable to standard upper endoscopy in its ability to diagnose BE, as well as dysplasia, with good inter-observer agreement [12]. These findings were further confirmed in a randomized, blinded, cross-over study [13]. One hundred twenty-one patients were randomized either to TNE followed by standard upper endoscopy or *vice-versa* and it was noted that the prevalence of BE in both groups was comparable (26% *vs*.** 30%; *P* = 0.503) but there was only moderate level of agreement between the two approaches (*k* = 0.591). Shariff *et al*.** performed a randomized, cross-over study comparing TNE with standard upper endoscopy in 95 patients and showed that the sensitivity and specificity of TNE for diagnosing BE were 0.98 and 1.00, respectively [14]. These studies also noticed better overall acceptance/tolerance of TNE over conventional endoscopy. Alashkar *et al*.** recently extended these observations, demonstrating that physician extenders can safely and accurately perform unsedated transnasal endoscopies in an outpatient setting, which can lead to still-lower screening costs [15]. Widespread use of TNE could cut down screening costs—especially if applied to high-risk patients—but widespread use will need greater acceptance by physicians and patients, more data on long-term outcomes, and cost-effectiveness studies.

### Cytosponge ^TM^

Non-endoscopic methods for diagnosing BE and EAC rely on collecting specimens without direct visualization and using various biomarkers to identify metaplasia and dysplasia. Cytosponge is a non-endoscopic sampling device that can be used to collect cell samples from the esophagus. Immunohistochemical staining (IHC) of these cells to detect Trefoil factor 3 (TFF3) helps in distinguishing BE cells from the gastric cardia and squamous cells of the esophagus and larynx [16]. The device consists of a 30 mm polyurethane sponge contained within a capsule that is attached to a string. After swallowing, the capsule dissolves in the stomach in 3–5 minutes and the sponge can be retrieved by pulling the string. While being pulled back, the sponge collects cells from the esophageal mucosa, which can be fixed in a cell-block and stained for TFF3. Kadri *et al*.** evaluated the acceptability and accuracy of Cytosponge in the primary care setting in a cohort study of 501 patients from the United Kingdom and showed that Cytosponge with TFF3 IHC had sensitivity and specificity of 73% and 94%, respectively, for diagnosing 1 cm or longer circumferential BE segments, which further increased to 90% and 93.5%, respectively, for circumferential BE segments 2 cm or longer [17].

The Barrett s Esophagus Screening Trial 2 (BEST2 Study) is a recently published case-control study that involved 1110 patients who underwent evaluation by both Cytosponge and endoscopy [18]. The Cytosponge was successfully swallowed by 94% patients and the overall sensitivity and specificity of Cytosponge in diagnosing BE of at least 1 cm circumferentially or at least 3 cm non-circumferentially were 80% and 92%, respectively. The study also showed an increase in sensitivity (i) with increase in the BE segment length and (ii) if the Cytosponge was swallowed twice during the study period. In a recent, comparative cost-effectiveness analysis using a micro-simulation model, it was shown that Cytosponge screening followed by treatment was more cost-effective than endoscopic screening and treatment, with an incremental cost-effectiveness ratio of $15.7 K *vs.* $22.2 K per QALY (quality adjusted life years), respectively [19]. The large size (3 cm) of the Cytosponge once open and contamination of the samples with oropharyngeal secretions and denuded mucosa are issues that need to be resolved. A prospective trial is currently under way in the United State to evaluate the acceptability and adequacy of this test (ClinicalTrials.gov. NCT02395471; not yet open for recruitment). Apart from the concerns about the acceptability of the currently available, large Cytosponge device, the reliance on biomarkers for diagnosing BE and EAC is another major limitation, since no specific biomarker has shown high sensitivity for diagnosing BE when compared with the ’gold standard’ histological diagnosis.

### Esophageal capsule endoscopy

The PillCam ESO capsule endoscope (Given Imaging Ltd., Yoqneam, Israel) is a dual-camera capsule endoscope specially designed for obtaining images of the esophagus. In a multi-center trial, esophageal capsule endoscopy (ECE) was done in 106 patients before endoscopic evaluation and it was found that ECE had sensitivity, specificity, positive predictive value (PPV) and negative predictive value (NPV) of 97%, 99%, 97% and 99%, respectively, in the diagnosis of BE [20]. It is important to note that histological confirmation was not used as the ‘gold standard’ for Barrett’s diagnosis in this study. These results were challenged by a subsequent study, in which 90 patients with histologically confirmed BE (by both screening and surveillance) underwent ECE followed by endoscopy. The sensitivity, specificity, PPV and NPV of ECE were 67%, 84%, 22% and 98%, respectively [21]. In a Veterans Administration study, the sensitivity, specificity, PPV and NPV for ECE diagnosis of BE in patients with gastroesophageal reflux disease (GERD) symptoms were 67%, 87%, 60% and 90%, respectively [22]. The sub-optimal sensitivity of ECE for BE diagnosis was confirmed by another study from France [23]. A meta-analysis of nine studies, which included 618 patients, evaluated the diagnostic accuracy of ECE for BE [24]. Using histological confirmation of intestinal metaplasia as the reference standard for BE diagnosis, the pooled sensitivity and specificity of ECE for BE diagnosis were 78% and 73%, respectively. Currently available data show that the sensitivity of ECE in diagnosing BE in patients undergoing screening is, at best, modest, hence ECE is currently not recommended for routine screening.

## Advances in surveillance: early diagnosis of high-grade dysplasia and adenocarcinoma

The latest published data have shown that the annual rate of progression to esophageal adenocarcinoma is around 0.1–0.3% [9, 10] and is lower than the previous estimate of 0.5% [25]. The incidence of adenocarcinoma in patients with high-grade dysplasia (HGD) approaches 6%, with one study reporting an incidence of 19%. Since these patients should undergo eradication therapy to prevent progression to cancer, efforts are being focused on improving the diagnostic yield for HGD.

Patients with non-dysplastic BE and low-grade dysplasia (LGD) should undergo regular surveillance to detect advanced histology that would benefit from eradication/definitive therapy. In these patients, the recommended intervals for surveillance are 3–5 years and 0.5–1 year, respectively. The AGA has established guidelines for a systematic biopsy protocol in BE patients undergoing surveillance. This ‘Seattle‘ protocol, which includes 4-quadrant biopsies every 1–2 cm of Barrett’s segment, along with separate sampling of areas of nodularity/mucosal irregularity, is aimed to detect HGD but has its limitations.

Strict adherence to the Seattle protocol is important since, in one study, only around 40% of high-grade dysplasia and esophageal adenocarcinomas were identified as endoscopically suspicious lesions locations during initial high-definition white-light endoscopy (WLE) [26]. Studies have shown that adherence to the recommended Seattle protocol is not very high and only 50% of endoscopists practicing in the community setting in the United States follow the recommended guidelines [27]. Similar results have been reported from the Netherlands [28]. Interestingly, in both studies the adherence to the protocol decreased with increasing length of the Barrett’s segment—which is worrisome, since longer BE segments have a higher incidence of advanced histology. Despite multiple biopsies, unsuspected intramucosal cancer (IMC) can be present in up to 40% of patients who undergo appropriate surveillance biopsies in accordance with the Seattle protocol and it might be no more effective at diagnosing HGD or IMC than a less-intensive surveillance protocol [29].

Given the poor adherence to BE surveillance and the inability to identify dysplasia, other markers of dysplasia—as well as endoscopic imaging techniques—are being studied. The use of biomarkers for detection of dysplasia is currently only in the investigational stages and is not recommended for routine, clinical decision-making. Various modalities—including chromoendoscopy, narrow-band imaging (NBI) with magnification and confocal laser endomicroscopy—are being studied to identify high-risk lesions during visual inspection. The AGA currently recommends detailed examination under WLE and these additional imaging techniques are not recommended (Table 1).

**Table 1.** Surveillance of Barrett’s esophagus

High-resolution white light endoscopy (bare minimum)
Optical chromoendoscopy
Narrow-band imaging (NBI)
Flexible spectral imaging color enhancement (FICE)
iScan
Confocal laser endomicroscopy (CLE)
Optical coherence tomography (OCT)
Dye based chromoendoscopy
Methylene blue
Indigocarmine
Acetic acid

Detailed examination under WLE should be performed in all patients undergoing surveillance for BE. High-definition endoscopes are available that capture images with up to 2.1 million pixels, compared with the standard-definition endoscopes that have up to 400 000 pixels. These newer high-definition endoscopes allow better resolution of the surface mucosa and can also magnify images 70–140 times, compared with 30–35 times magnification with standard-definition endoscopes [30]. There are currently no randomized trials comparing WLE examination with standard- and high-definition endoscopes for detection of advanced histology in BE, but studies using high-definition endoscopes with chromoendoscopy and NBI have shown positive results; hence the consensus statement published in 2012 recommended against the use of standard-definition endoscopes and suggested that high-definition scopes should be used for surveillance of Barrett’s epithelium, although it acknowledged the lack of conclusive data to support this recommendation [31]. Despite the use of high-definition scopes, the Seattle protocol, with random four-quadrant biopsies every 1–2 cm, should be followed. The time spent inspecting BE is another important factor in detecting advanced lesions; Gupta *et al*.** compared Barrett’s inspection time of less than 1 minute per cm of BE against a period of more than 1 minute per cm using high-definition WLE examination and found that more endoscopically suspicious lesions (54.2% *vs.*13.3%; *P* = 0.04) and HGD/EAC (40.2% *vs*.** 6.7%; *P* = 0.06) were detected with longer inspection time [32].

### Dye-based chromoendoscopy

Dye-based chromoendoscopy involves spraying a chemical solution on the mucosa to enhance visualization of the mucosal surface and vascular pattern by differential absorption. Various dyes that have been studied for enhanced imaging of BE include methylene blue, acetic acid and indigo carmine.

Methylene blue is a vital stain that is preferentially absorbed by the intestinal and colonic mucosa and hence can differentiate intestinal metaplasia from the normal squamous mucosa. Initial studies using methylene blue showed that application of this substance increased the detection of intestinal metaplasia as well as advanced histology (HGD and IMC) [33–36], but other studies showed no incremental yield from its use [37, 38]. Two meta-analyses addressed this issue and neither found any benefit of methylene blue over random four-quadrant biopsies [39, 40]. Similarly, both indigocarmine—a contrast stain—and acetic acid—a contrast-enhancing agent—have shown promising results in improving the detection of advanced histology [33, 41–46], but dye-based chromoendoscopy is currently not recommended for routine use in surveillance of Barrett’s epithelium. Further long-term data and multi-center randomized trials are needed before these modalities are widely accepted.

Over the last few years, the use of acetic acid has attracted renewed interest in detecting advanced histology in patients undergoing surveillance for BE. Acetic acid has been used for a long time to detect cervical intra-epithelial neoplasia during colposcopy; after its application, acetic acid initially causes an acetowhitening effect, which masks the submucosal capillaries and makes the mucosal surface prominent by rendering it more opaque, thus enhancing the surface pit-pattern and allowing for detailed examination of the mucosa. The loss of acetowhitening is characterized by mucosal reddening and swelling. Dysplastic areas lose acetowhitening faster than non-dysplastic areas, which again helps in the identification of dysplasia [47, 48]. Guelrud *et al.* were the first to use acetic acid to detect residual islands of BE in 21 consecutive patients after endoscopic therapy [49]. Application of acetic acid showed remnant BE islands in 11 patients (52%), which were not seen on conventional endoscopy.

Multiple studies used acetic acid with magnification for identification of intestinal metaplasia, but the first prospective study to detect advanced histology with acetic acid without magnification was done by Vasquez-Iglesiaz *et al*.** in 2007 [50]. They sprayed 3% acetic acid and then obtained guided biopsies in one hundred patients with normal endoscopic appearance and showed that sensitivity and specificity for identification of dysplasia and EAC using acetic acid were 100% and 98%, respectively. Longcroft *et al*.** compared WLE and protocol-based biopsies with acetic acid and targeted biopsies (both modalities without magnification) in 190 endoscopies and showed that acetic acid chromoendoscopy had a sensitivity and specificity of 95.5% and 80%, respectively, for diagnosis of neoplasia (included low-grade dysplasia, high-grade dysplasia and early cancer) [44]. They also showed that acetic acid spray improved detection of BE neoplasia 2.5-fold compared with WLE alone (*P* = 0.001). The largest retrospective series comparing high-resolution WLE and acetic acid chromoendoscopy without magnification was recently published from the United Kingdom: Tholoor *et al*.** compared 627 patients with BE who underwent 972 procedures for surveillance either with WLE and protocol biopsies or acetic acid-guided targeted biopsies [43]. They showed that overall neoplasia detection rates were significantly higher in the acetic acid group than in the WLE group (12.5% *vs*.** 2%; *P* = 0.001). This difference was significant across all grades of neoplasia (LGD, HGD and superficial cancers). On subgroup analysis of all patients with neoplasia, it was found that acetic acid-targeted biopsies detected 87% of the dysplastic lesions, compared with 30% with standard biopsy protocol (*P* < 0.001). Acetic acid chromoendoscopy has also shown to be a more cost-effective approach (based only on pathology costs) compared with WLE and standard biopsy protocol [45]. Its relative safety when compared with other dyes, absence of any known carcinogenic effects and the low cost of acetic acid make it an attractive tool for BE surveillance, although further long-term prospective studies with improved outcomes are needed.

### Optical chromoendoscopy

Optical chromoendoscopy involves detailed examination of the mucosal surface and vascular pattern by using filters of different wavelengths, image processing and magnification. As mentioned above, with the availability of high-definition endoscopes, high-resolution WLE is the bare minimum for evaluation of BE and is recommended by different gastroenterology societies.

NBI is the most commonly available and most-studied optical chromoendoscopy modality. Studies have shown that NBI is superior to standard definition WLE in detecting dysplasia in BE [51] but studies comparing high-resolution WLE with NBI have not shown superiority of NBI for surveillance purposes [52, 53]. In one of the earlier studies, Sharma *et al*.** graded NBI images based on mucosal and vascular pattern and showed that the presence of an irregular/distorted pattern on NBI had a sensitivity, specificity and PPV of 100%, 98.7% and 95.3%, respectively, for diagnosing HGD; however this study was not blinded and there was no control group [54]. Studies using NBI with magnification have also reported great success in diagnosing advanced histology and a simplified classification of various surface patterns, to diagnose different histological grades of BE based on NBI with magnification, has been proposed [55]. Mannath *et al*.** performed a meta-analysis of eight studies, including 446 patients with 2194 lesions, to evaluate the diagnostic accuracy of NBI with magnification in identifying specialized intestinal metaplasia and HGD [56]; they showed that sensitivity, specificity, diagnostic odds ratio and area under the curve for HGD diagnosis using NBI with magnification were 0.96, 0.94, 342.5 and 0.99, respectively, based on per-lesion analysis.

In an international, multicenter, cross-over study, Sharma *et al*.** compared NBI and WLE without magnification in 153 patients undergoing endoscopy for screening or surveillance of BE [57]; they showed that there was no difference in the proportion of lesions with advanced histology (HGD and EAC) detected by either WLE or NBI (4% *vs*.** 7%; *P* = 0.1). It is important to note that only 14 out of 153 patients in this study had advanced histology. In a recent meta-analysis, Song *et al*.** concluded that NBI detects HGD dysplasia with a per-patient sensitivity and specificity of 0.91 and 0.95, respectively, with corresponding per-lesion values of 0.69 and 0.90, respectively [58]; but the studies that evaluated diagnostic accuracy of NBI for HGD were heterogeneous as they included studies with NBI alone, as well as those that used NBI with magnification. To conclude, NBI with magnification probably diagnoses advanced histology with a higher yield than high-resolution WLE, but the same cannot be conclusively said for NBI without magnification. Although not recommended by societal guidelines, NBI with magnification-targeted biopsies should be obtained when available, in addition to the high-resolution WLE examination and the Seattle biopsy protocol for surveillance of BE.

### Confocal laser endomicroscopy

Confocal laser endomicroscopy (CLE) is based on the illumination of a fluorescent target by a low-powered argon ion laser (488 nm wavelength) and detection of light emanating from that target by a photodetection device after it passes through a pinhole, followed by image processing [59]. It allows the highly detailed evaluation of surface epithelium, as well as the vascular pattern of serial sections of thick *in vivo* specimens. CLE can be performed either by using endoscopes with an integrated confocal imaging capability (Pentax, Tokyo, Japan) or by using a CLE probe advanced through the accessory channel of endoscopes (CellVizio, Mauna Kea Technologies, Paris, France). In one of the earlier studies, Kiesslich *et al*.** used CLE in 63 patients undergoing endoscopy for long-standing reflux or Barrett’s surveillance and showed that CLE has sensitivity and specificity of 98.1% and 94.1%, respectively (PPV 97.2%, NPV 96% and accuracy 96.8%). They also showed that CLE was useful in predicting advanced histology (LGD, HGD and EAC) with sensitivity, specificity, PPV, NPV and diagnostic accuracy of 92.9%, 98.4%, 92.9%, 98.4% and 97.4%, respectively. In the first prospective, randomized, blinded, cross-over trial of CLE *vs.* standard biopsy protocol, Dunbar *et al*.** evaluated 39 patients undergoing surveillance for BE (16 with suspected HGD or EAC and 23 for surveillance only) and showed that CLE had higher diagnostic yield for advanced histology (HGD or EAC, 33.7% *vs*.** 17.2%; *P* = 0.01) with a lower mean number of mucosal biopsies needed (9.8 *vs*.** 23.7; *P* = 0.002) in the high-risk group [60]. They also showed that two-thirds of patients in the surveillance group did not require any biopsies at all. This study was done using endoscopes with CLE capability.

Multiple studies evaluated probe-based CLE (pCLE) for the diagnosis of BE and advanced histology in BE, but were limited either by sample size [61] or by low diagnostic accuracy [62, 63], although some showed promising results as well [64, 65]. In an effort to improve the diagnostic accuracy of pCLE, Gaddam *et al*.** first proposed criteria to predict advanced histology and then tested the accuracy of these in a two-phase study [66]. The criteria proposed included (i) saw-tooth appearance of epithelial surface, (ii) goblet cells not easily identified, (iii) unequal distance between glands, (iv) unequal size and shape of glands, (v) presence of enlarged cells and (vi) presence of irregular cells that are not equidistant. Using these criteria, the overall accuracy, sensitivity, specificity, PPV and NPV for the diagnosis of advanced histology (HGD and EAC) were 82%, 76%, 85%, 76% and 85%, respectively. Two factors that increased the accuracy were “the endoscopist’s confidence about the diagnosis” and high-quality video images. They also showed there was no difference between experienced and inexperienced physicians in the overall diagnostic accuracy, supporting the objective nature of the proposed criteria.

In a randomized, controlled, multicenter trial, Canto *et al*.** compared high-definition WLE and random biopsies with a combination of high-definition WLE, endoscope-based CLE (eCLE) and targeted biopsies [67] in 192 patients with BE. They showed that the combination of eCLE with high-definition WLE increased the sensitivity for detection of neoplasia from 40% to 96% (*P* < 0.0001); also the combination of high-definition WLE + eCLE + targeted biopsies significantly increased the diagnostic yield for neoplasia over targeted biopsies WLE with standard biopsies (34% *vs*.** 7%; *P* < 0.0001). In a meta-analysis involving seven studies (345 patients and 3080 lesions), it was shown that CLE had sensitivity and specificity of 68% and 88% for the diagnosis of HGD and early EAC, based on per-lesion analysis [68]. These data suggest that CLE is not yet ready for prime time and might not be the main surveillance modality for BE in future, but might have a role in selected patients who have advanced histology on random biopsies but no identifiable lesions on high-resolution WLE. Still, the high cost of CLE, the need for intravenous contrast and the availability of more affordable modalities such as NBI might limit its widespread use.

### Optical coherence tomography/volumetric laser endomicroscopy

Optical coherence tomography (OCT) is a relatively new imaging modality based on interferometry. It involves the use of a light signal to obtain cross-sectional images in high resolution, by measuring the path length of reflected light followed by image processing. It offers very high spatial resolution of the order of 1–15 µm [69]. Many reports described the feasibility of OCT for high-resolution *in vivo* imaging of the gastrointestinal tract, especially the esophagus [70–73]. Poneros *et al*.** showed that OCT had high sensitivity (97%) and specificity (92%) in the diagnosis of specialized intestinal metaplasia [74]. Isenberg *et al*.** performed the first prospective study to evaluate the use of OCT in diagnosing advanced histology (LGD, HGD and EAC) in 33 patients undergoing surveillance for BE [75]. They used probe-based OCT and showed that the sensitivity, specificity, PPV, NPV and overall diagnostic accuracy for detection of advanced histology were 68%, 82%, 53%, 89% and 78%, respectively. Along with relatively low sensitivity, the study also showed wide inter-observer variability in diagnostic accuracy between four endoscopists (range 56–98%). Evans *et al*.** developed a scoring system or ’dysplasia index’ (score range 1–4) based on surface maturation and gland architecture and used this to prospectively evaluate 242 biopsy-correlated images from 55 patients in a blinded fashion [76]. They showed that a dysplasia index score of 2 or higher was 83.3% sensitive and 75% specific for the diagnosis of HGD/IMC, with excellent inter-observer agreement (*k* = 0.89). Since then there have been major advances in the OCT equipment available, including high-resolution imaging, three-dimensional imaging capability and development of balloon catheters with translation of the OCT imaging probe to evaluate a larger area in less time [77–81].

The Nvision Volumetric Laser Endomicroscopy (VLE) Imaging System (Nine Point Medical, Bedford, MA, USA) was approved by the US Food & Drug Administration (FDA) in 2012 and employs OCT to perform 3 mm-deep cross-sectional scans over a length of 6 cm using balloon catheters, at a very high resolution of 7 µm in real time, thus making it a volumetric imaging system. A recent multi-center, prospective study involving 100 patients showed that VLE was feasible using the Nine Points Medical system [82], but further longitudinal studies are needed to study its diagnostic accuracy and its role in the management of BE. A niche use proposed for OCT, due to its ability to provide deeper imaging, is the detection of buried Barrett’s glands after ablative therapy [83, 84].

## Advances in treatment of barrett’s esophagus and early esophageal adenocarcinoma

Treatment options for BE focus on the prevention of EAC and definitive therapy of early neoplasia once detected. As discussed above, dysplasia is currently the best available clinical marker for predicting the development of EAC. High-grade dysplasia has the highest risk of progression to EAC and, hence, consensus guidelines from different GI societies recommend endoscopic eradication of HGD. The incidence of EAC is very low in patients with LGD (0.1% per year) and hence most societies recommend routine surveillance in patients with confirmed BE and LGD. A major limitation in the management of LGD is the poor inter-observer agreement between pathologists in its diagnosis, leading many to believe that the true incidence of progression to advanced histology is underestimated by labeling non-dysplastic BE as LGD. Curvers *et al*.** reviewed pathology specimens in 147 patients who had been diagnosed with LGD, and showed that 85% were downgraded to non-dysplastic BE after consensus review by two expert GI pathologists [85]. In the remaining 15% patients with consensus-confirmed LGD, the rate of progression to advanced histology (HGD or EAC) was 13.4% per year. In a multi-center, randomized trial, it was shown that the risk of progression to HGD/EAC was reduced by a significant 25% in patients undergoing radiofrequency ablation for LGD (1.5% *vs*.** 26.5%; *P* < 0.001) [86]. The lower risk of progression to advanced histology after ablative therapy for LGD was also shown in a retrospective study from three medical centers in the United States [87]. These findings underscore the importance of accurate diagnosis of LGD and the need to be certain about the diagnosis before considering ablation in these patients.

There is agreement that all patients with HGD should undergo definitive ablative therapy. At this time, endoscopic treatment has entirely replaced surgery for the management of HGD and IMC. The different endoscopic modalities available for HGD include ablation by superficial tissue injury [radiofrequency ablation (RFA), cryotherapy, argon plasma coagulation (APC), and photodynamic therapy] or endoscopic resection [endoscopic mucosal resection (EMR) and endoscopic submucosal dissection (ESD)] and are listed in Table 2. Photodynamic therapy and APC have both shown good results but, because of side-effects with the former and technical challenges in treating a large segment of BE with the latter, both have been largely replaced by RFA and cryotherapy.

**Table 2.** Treatment options for Barrett’s esophagus

Ablation (without obtaining tissue)
Radiofrequency ablation (RFA)
Cryotherapy
Argon plasma coagulation (APC)
Photodynamic therapy
Resection-based strategies
Endoscopic mucosal resection (EMR)
Endoscopic submucosal dissection (ESD)
Hybrid Therapy

### Radiofrequency ablation

**Figure 1.**
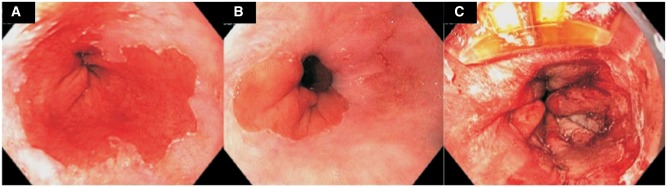
Radiofrequency ablation (RFA) of Barrett’s esophagus. (**A**) A 62 year-old male patient with a short segment of Barrett’s esophagus and flat high-grade dysplasia. (**B**) Residual Barrett’s segment after a single session of circumferential RFA treatment. (**C**) Focal RFA of the residual Barrett’s segment.

**Figure 2.**
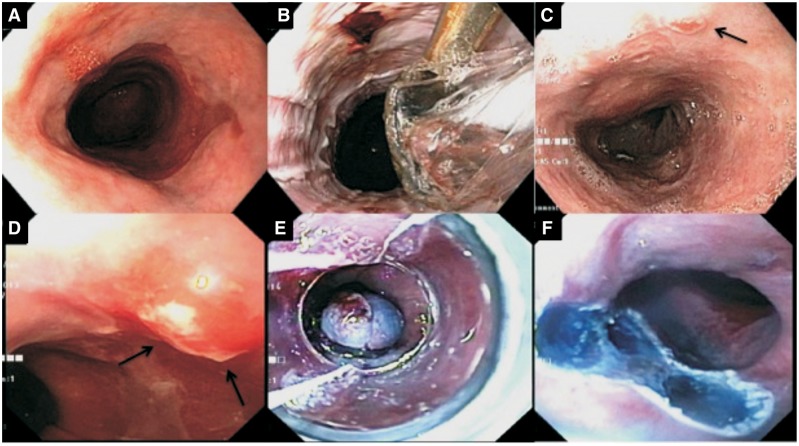
Hybrid therapy for Barrett’s esophagus. (**A**) A 71-year-old male with long-segment Barrett’s esophagus (Prague C8M9). (**B**) Circumferential radiofrequency ablation (RFA) using a balloon RFA catheter was performed. (**C** and **D**) Repeat endoscopy at 6 months after two sessions of RFA showed neosquamous epithelium and a small nodule (arrows). (**E**) Successful endoscopic mucosal resection (EMR) of the nodule was performed using a band ligation and snare resection technique, after injecting with indigocarmine. (**F**) The mucosal defect after resection showed intact submucosa stained with indigocarmine. Focal RFA of the residual Barrett’s was also performed at this session.

RFA involves thermal injury to the superficial 0.5 mm of the esophageal mucosa, resulting in tissue necrosis and eventual growth of neo-squamous mucosa (Figures 1 and 2). Multiple RFA devices are available and include esophageal-diameter non-endoscopic balloons, over-the-scope catheters and through-the-scope catheters, depending on the size of the BE segment. The short-term efficacy for complete eradication of intestinal metaplasia and complete eradication of dysplasia after RFA is excellent and has been confirmed by multiple studies including the landmark Ablation of Intestinal Metaplasia containing dysplasia (AIM dysplasia) trial. [88–90]. The AIM dysplasia trial is a multicenter, randomized, sham-controlled trial that studied the outcomes of RFA in BE patients. In their initial study, the researchers randomized 127 patients with dysplastic BE in a 2:1 ratio to either RFA ablation or a sham procedure and showed that, at 12-months follow-up, complete eradication of dysplasia was achieved in 81% of patients with RFA, compared with only 19% patients in the sham group (*P* < 0.001) [88]. Follow-up of the AIM dysplasia trial patients (cross-over design) showed excellent long-term results after RFA, with the rates of complete eradication of intestinal metaplasia and dysplasia approaching 89% and 93%, respectively, after two years in patients with HGD [91]. Another study from Europe also showed 90% remission at 5-year follow-up after RFA for BE with HGD [92], but other studies have shown a significant rate of recurrence of BE after RFA on long-term follow-up, ranging from 20–33% at two years [93, 94]. It is important to note that the majority of recurrent BE is non-dysplastic (78–86%) and can be easily treated endoscopically; however, this highlights the importance of close surveillance in these patients after RFA. Older age, longer length of BE segments and non-Caucasian race have been associated with higher rates of recurrence. The studies mentioned above have shown a favorable side-effect profile for RFA, with stricture rates ranging from 4–12%, which can be treated successfully with endoscopic dilation. Amongst all ablative options available for BE, the long-term data for efficacy and safety are most conclusive for RFA, making it the currently favored and most common ablative modality used for the ablation of Barrett’s esophagus.

### Cryotherapy

Cryotherapy is a relatively new technology of non-contact tissue ablation and involves snap-freezing of the surface epithelium—either by using liquid nitrogen or rapidly expanding carbon dioxide—leading to immediate cell death. Cell injury occurs during reperfusion by generation of free radicals in the frozen tissue as it is re-oxygenated. Johnston *et al*.** published the first pilot study describing the use of cryotherapy in eleven patients with BE. They showed that histological reversal of BE was noted in 78% (9/11) after 6-month follow-up without any major complications [95]. Since then, other retrospective and smaller prospective studies have shown the efficacy of cryotherapy, with remission of HGD achieved in 94–97% of the patients and complete eradication of intestinal metaplasia in 53–81% patients [96–98]. In the largest available prospective, multicenter trial of cryotherapy for BE, 91% of the patients benefited from eradication of their HGD, 81% experienced eradication of all dysplasia and 65% achieved eradication of all intestinal metaplasia after a mean follow-up period of 21 months and an average of 3.5 spray cryotherapy sessions [99]. There was no perforation or mortality, although two patients developed bleeding and stricture (one each). Although these trials were not controlled, the reported success rates were similar to those of RFA. Unlike RFA, cryotherapy can be applied to nodular areas, in the presence of bleeding, and may cause less fibrosis; however, there is a relative lack of long-term data about the efficacy of cryotherapy compared with RFA, and there is no head-to-head comparison of the two ablative modalities.

### Buried metaplasia

Buried Barrett’s metaplasia (‘buried Barrett’s’) refers to the presence of metaplastic glands in the *lamina propria,* with overlying normal-appearing squamous epithelium. Initially described in patients with BE without ablation, it was thought to be present in the areas where BE abuts the squamous epithelium. Buried Barrett’s has now been described in areas of neosquamous epithelium, which develop after ablative therapy. Although the clear significance of buried Barrett’s is not completely known, there have been case reports of development of EAC in the buried metaplasia, mainly after argon plasma coagulation of BE [100, 101]. The exact incidence is not fully known but, with advances in imaging and the development of surveillance guidelines, more cases of subsquamous metaplasia are being reported. Kohoutova *et al*.** reported a case series of 288 patients treated with either PDT or RFA from 1999 to 2014 and found that subsquamous neoplasia was diagnosed in seven patients (2%) [102]. A systematic review attempted to address the issue of the true incidence of buried metaplasia. Gray *et al*.** reviewed five studies that reported a baseline incidence ranging from 0–28% in BE patients [103]. They also reviewed 18 studies, with a total of 1004 patients who underwent biopsies after RFA, and found an incidence of only 0.9% after RFA but it should be noted that the adequacy of regular biopsies to identify this entity is probably not high, since most biopsy specimens of neosquamous epithelium do not include *lamina propria*, which is the location for the buried metaplasia. Published data indicate that the adequacy of biopsies during surveillance is very variable and the percentage of surveillance biopsies that sample *lamina propria* ranges from 13–90% [103]. Even though, theoretically, buried metaplasia has neoplastic potential, the real-life incidence of the buried metaplasia, as well as the incidence of advanced histology in these buried glands, is still not fully known. Recent reports have described how both VLE and OCT can help in the detection of subsquamous metaplasia [79, 83, 84, 104], but the data are still limited and further studies are needed to fully understand the clinical significance of buried metaplasia, effective diagnostic strategies and how to treat it.

### Endoscopic mucosal resection

Visible nodules or lumps in the metaplastic BE have a high incidence of invasive cancer, with reports indicating that 40–78% of visible lesions can harbor cancer [105–107]. Regular biopsies from these lesions underestimate the degree of dysplasia in these lesions. Along with being an effective endoscopic treatment modality, endoscopic mucosal resection (EMR) offers significant advantage in histological staging and predicting the depth of invasion. Multiple studies have shown that EMR leads to better inter-observer agreement on the degree of dysplasia, and can lead to change in the final histological stage when compared with biopsy specimens in up to 49% of the patients [107–110]; hence, due to higher incidence of advanced histology and also better staging, EMR of all visible nodules or lesions within the metaplastic BE is recommended. Hybrid therapy that involves EMR of visible lesions, followed by endoscopic ablation of metaplastic epithelium (Figure 2), has been shown to be an effective strategy for the management of BE [111–113]. Harrero *et al*.** studied hybrid therapy (EMR of visible lesions followed by RFA) in 26 patients with BE segments longer than 10 cm and showed that complete remission of neoplasia and intestinal metaplasia were achieved in 83% and 79% of patients, respectively, after a mean follow-up period of 29 months, without any severe complications or significant recurrence of neoplasia [114]. Some studies have raised concerns about decreased efficacy and increased stricture rate of RFA after EMR, but a recent analysis of patients from the United States Radiofrequency Ablation Registry (US RFA) showed that preceding EMR neither decreased the efficacy nor increased the complication rates of RFA, as compared with RFA alone without preceding EMR [115].

Despite the excellent outcomes of hybrid therapy, there has been some concern about a high incidence of synchronous and metachronous HGD, as well as recurrent high-grade lesions after focal EMR (14–24%) [116–118]. Seewald *et al*.** proposed circumferential EMR and complete removal of BE (complete Barrett’s eradication with EMR, CBE-EMR) in patients with either multifocal high-grade lesions or IMC detected on random biopsies but not easily identified on endoscopic examination [119]. Since then, various studies have shown the efficacy of CBE-EMR for HGD and early mucosal cancer, with the complete remission rate of intestinal metaplasia ranging from 76–95% but with high incidence of symptomatic strictures approaching up to 49% [120–124]. In a recent study, Konda *et al*.** reviewed their experience of CBE-EMR in 107 patients with BE and HGD or IMC [125]. They showed that, after a mean follow-up of 40.6 months, BE was eradicated in 80.4% and 98.8% patients—based on intention-to-treat and per-protocol analyses, respectively—but strictures and symptomatic dysphagia were noted in 41% and 37% patients, respectively. A recent systematic review showed that RFA and complete EMR were equally effective in the short-term treatment of BE but that complete EMR was associated with higher rates of complications [126]; hence, complete EMR should be considered only in patients with multi-focal high-grade dysplasia or IMC and in those patients in whom incidental IMC is noted on random biopsies, but the lesion cannot be identified with certainty on endoscopic examination. Hybrid therapy with EMR of visible lesions and ablation of remaining HGD should be the treatment of choice for HGD in the majority of patients, due to its proven high efficacy and favorable safety profile.

## Management of early esophageal adenocarcinoma

**Figure 3.**
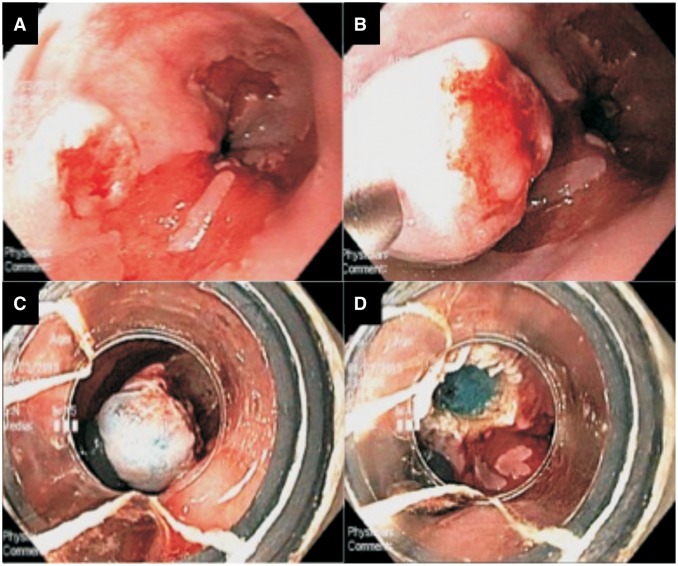
Endoscopic mucosal resection (EMR) for early esophageal adenocarcinoma. (**A**) A nodular lesion with central depression causing concern about malignancy at the proximal end of Barrett’s segment. (**B**) The lesion lifted well with submucosal injection. (**C** and **D**) EMR was successfully performed using band ligation. The pathology showed intramucosal cancer (T1a) without any involvement of the deep and lateral margins.

Local ablative therapy is adequate for HGD and IMC, because the risk of lymph node involvement in these conditions is very low. Published studies have shown that up to 50% of stage T1b lesions (those involving the submucosa) can have lymph node involvement, hence surgical treatment is recommended [127, 128]. Dunbar *et al*.** reviewed 70 publications involving 1874 patients who underwent esophagectomy for HGD or IMC and showed that lymph node metastasis was found in 1.9% of patients with IMC and none of the 524 patients with HGD had lymph node involvement [129]. Due to the limited accuracy of EUS in differentiating T1a from T1b lesions, the most reliable predictor is histology and both EMR and ESD provide adequate tissue for this (Figure 3). The majority of the studies of EMR have included both HGD and EAC and studies of EMR exclusively for EAC are therefore limited. In one of the earliest studies, Ell *et al*.** retrospectively reviewed their data on EMR in 100 patients with Barrett’s EAC with low-risk criteria. These were defined as (i) lesion diameter <20 mm and macroscopically type I, IIa, IIb or IIc lesions < 10 mm, (ii) well- or moderately differentiated tumors and (iii) lesions limited to the mucosa and absence of lymphovascular involvement. They did not notice any major complications,and complete remission was achieved in 99% of patients, with a 5-year survival of 98%. Recurrence was noted in 11% of patients after a mean follow-up period of 36.7 months and was in all cases amenable to endoscopic therapy [130]. The same group recently published their data on 1000 patients who underwent EMR for mucosal esophageal cancer and showed that 96.3% had clinical remission after a mean follow-up period of 56.6 months. Surgery was needed for failed endoscopic therapy in only 3.7% of patients with a 10-year survival of 75% [131]. Again it is important to note that incidence of metachronous lesions in this study was 14.5%, hence regular surveillance after EMR is very important. Studies comparing EMR with esophagectomy for T1a cancer have shown comparable survival in both groups [132].

ESD was initially developed for endoscopic treatment of gastric cancers in Japan and provides the benefit of *en-bloc* resection. ESD has been successfully used for treatment of early esophageal squamous cell cancer but the data about ESD in Barrett’s neoplasia is limited. Recent studies from Asia and Europe have shown that ESD can achieve *en-bloc* resection in 90–100% patients with early EAC, with R0 resection achieved in 64–85% patients [133–135]. But ESD is a technically challenging procedure with high complication rates and post-ESD stricture rates approaching up to 60% [134]. ESD might in the future be of value in selected patients with early cancer that cannot be treated with EMR due to scarring from prior resection attempts or ablative treatment, but current data suggest that EMR of visible lesions and T1a adenocarcinoma is associated with good outcomes, without the complications associated with ESD and, hence, should be the favored treatment approach.

*Conflict of interest statement:* Amitabh Chak, MD holds patent applications for investigational balloon device and biomarkers for diagnosis of Barrett’s esophagus, none of which are discussed in this article.
